# Risk factors associated with cutaneous anthrax outbreaks in humans in Bangladesh

**DOI:** 10.3389/fpubh.2024.1442937

**Published:** 2024-10-15

**Authors:** Sukanta Chowdhury, Md. Saiful Islam, Najmul Haider, Muhammad Belal Hossain, Md. Ashraful Alam, Md. Ahmad Raihan Sharif, M. Salim Uzzaman, Mahbubur Rahman, Mahmudur Rahman, Farhana Haque

**Affiliations:** ^1^International Centre for Diarrhoeal Disease Research, Dhaka, Bangladesh; ^2^School of Population Health, Faculty of Medicine and Health, University of New South Wales, Sydney, NSW, Australia; ^3^School of Life Sciences, Faculty of Natural Sciences, Keele University, Keele, Staffordshire, United Kingdom; ^4^Department of Ecology and Evolutionary Biology, University of Tennessee, Knoxville, TN, United States; ^5^Institute of Epidemiology, Disease Control and Research (IEDCR), Ministry of Health and Family Welfare, Dhaka, Bangladesh; ^6^EMPHNET, Dhaka, Bangladesh; ^7^UK Public Health Rapid Support Team (UK PHRST), Department of Infectious Disease Epidemiology and Dynamics, London School of Hygiene and Tropical Medicine (LSHTM), London, United Kingdom

**Keywords:** anthrax, human, risk factors, Bangladesh, outbreak

## Abstract

**Objectives:**

To determine the risk factors associated with cutaneous anthrax infection in humans.

**Methods:**

During 2013–2016, we investigated total 26 anthrax outbreaks across the country. We additionally conducted a case–control study to identify risk factors by recruiting four controls for each enrolled case. Adjusted odds ratios (aOR) and 95% confidence intervals (CI) were estimated to identify risk factors using multivariate logistic regression.

**Results:**

Over the study period, a total of 1,210 suspected cutaneous anthrax cases were identified in seven districts of Bangladesh. Most of the cases (61%, *n* = 744) were detected from Meherpur district. Cases were detected over the year, with the peak number of outbreaks occurring in May. The overall attack rate of suspected cutaneous anthrax cases for 16 outbreaks was 20%, with the highest rate occurring among individuals aged 40–49 years. Persons who had a cut injury (aOR 19.04, CI: 4.08–88.86), weighed raw meat (aOR 5.73, CI: 3.03–10.83), mixed bones and meat (aOR 4.64, CI: 3.03–7.09), observed livestock slaughtering (aOR 2.86, CI: 2.02–4.04), had direct contact to an anthrax suspected livestock (aOR 2.68, CI:1.61–4.45), slaughtered livestock (aOR 2.29, CI: 1.3–4.02), and who did not wash hands with soap and water after direct contact (aOR 2.57, CI: 1.89–3.5) were more likely to develop cutaneous anthrax than people who did not have these exposures.

**Conclusion:**

Prior cut injuries on exposed body areas during meat handling and slaughtering of sick livestock were identified as potential risk factors for cutaneous anthrax, highlighting the importance of preventing the slaughter of sick animals. However, stopping slaughtering sick livestock, handling meat and livestock by-products to reduce anthrax exposures from livestock to humans may be difficult to achieve given the associated financial incentives in Bangladesh. Interventions such as hand washing with soap during slaughtering and processing meat can be targeted to affected communities to ameliorate some risk.

## Introduction

Anthrax is an acute bacterial zoonotic disease caused by *Bacillus anthracis* that infects livestock and human (1). *Bacillus anthracis* spores persist in soil for periods ranging from years to decades (2). Domestic cattle, sheep and goats often get infected after ingesting these spores from environment while grazing in pastures or through ingesting feedstuffs including concentrated feeds and grass that are contaminated with anthrax spores (3, 4). Anthrax usually causes three clinical forms of disease in humans: cutaneous, gastrointestinal, and inhalation anthrax. Of these three forms in humans, cutaneous anthrax comprises >95% of naturally occurring infections (5–8). In addition to these three distinct forms, a new type of anthrax known as ‘injectional anthrax’ has been reported in Europe (9). Humans contract cutaneous anthrax through direct skin contact with *B. anthracis* infected animals during slaughtering animals and/or processing animal by-products (10, 11). There was limited or inconclusive evidence regarding the transmission of *B. anthracis* between individuals or among animals (11).

In Bangladesh, livestock and human infections with *Bacillus anthracis* have been reported since 1986 (12). While outbreaks were consistently reported in livestock since at least the 1980s, only sporadic human infections were reported prior to 2009 (12, 13). Between 2010 and 2017, repeated outbreaks of human cutaneous anthrax from 16 districts resulted in a total of 2,581 suspected anthrax cases (14). Persons slaughtering sick livestock, handling raw meat, handling of sick livestock, living in proximity to slaughtering or livestock death sites and handling of skins of infected livestock were at higher risk for cutaneous anthrax infection (12, 15, 16). Outbreak investigations followed by a subsequent case–control analysis may be useful to identify significant risk factors. The objectives of the outbreak investigations and subsequent case–control study were to describe the magnitude of cutaneous anthrax in terms of person, place and time in Bangladesh; and identify the risk factors for acquiring cutaneous anthrax among the people who had exposure to raw meat, body fluids, carcass and/or by-products of sick or slaughtered or dead livestock potentially infected with anthrax.

## Methods

### Study area

**Figure 1:**
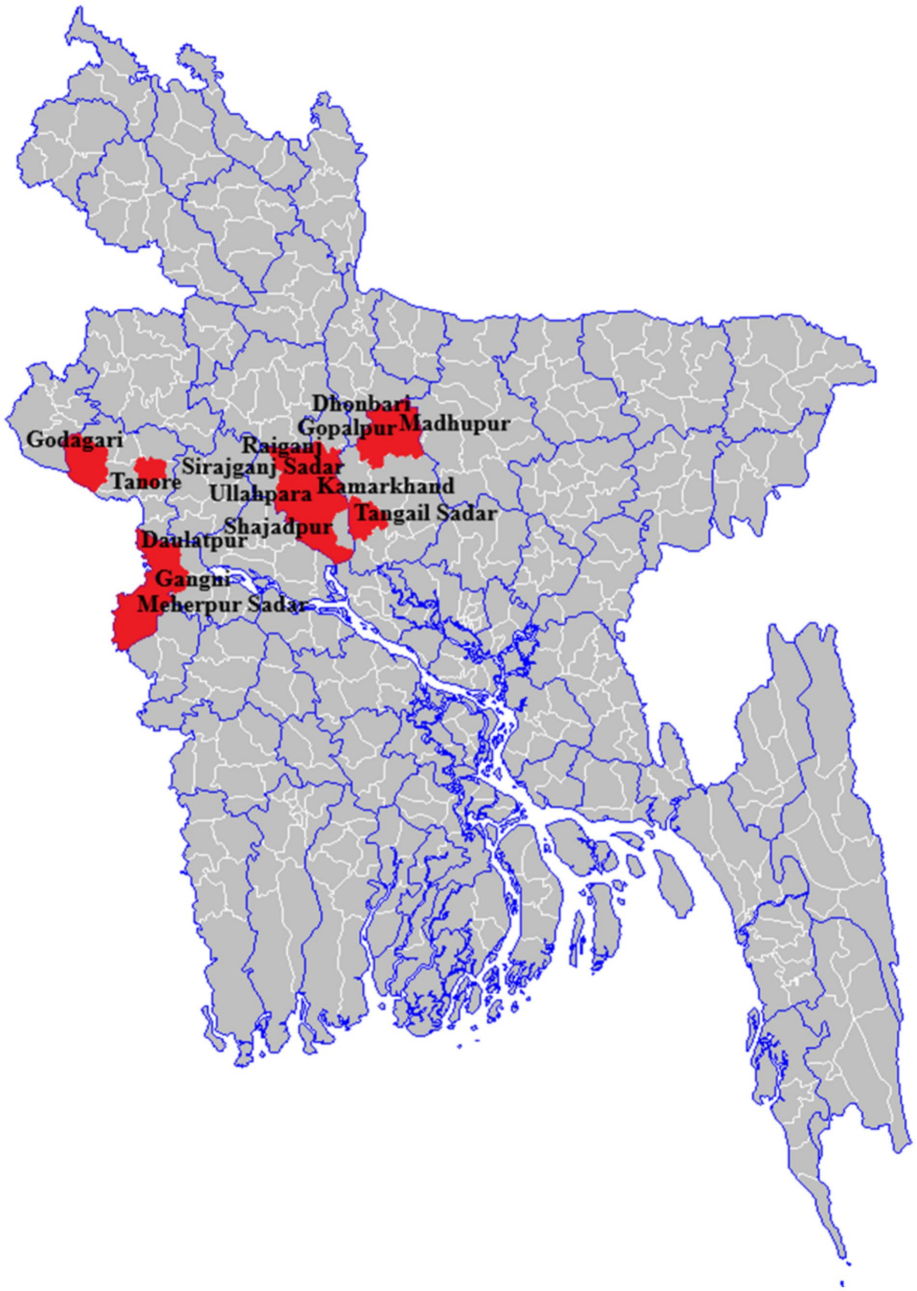
Anthrax affected seven districts that reported at least one cutaneous anthrax outbreak, 2013–2016, Bangladesh (*n* = 1,210).

Outbreak investigations were conducted as part of the national anthrax surveillance in humans from 2013 to 2016. The Institute of Epidemiology, Disease Control and Research (IEDCR) under the Directorate General of Health Services (DGHS), Bangladesh led and coordinated the sentinel surveillance for human anthrax. The surveillance team visited affected communities in Bangladesh where suspected cutaneous anthrax case(s) were reported within 4 weeks of exposure to raw meat, body fluids, carcass or by-products of suspected livestock case(s) (Figure 1).

### Outbreak notification and case identification

Human cases clinically compatible with symptoms of suspected cutaneous anthrax were reported to the IEDCR through any of the government health channels including district and sub-district healthcare managers, a hotline, a web-based integrated disease surveillance or a media-based surveillance (Supplementary Figure 1).

A multi-disciplinary One Health team consisting of trained epidemiologists, physicians, veterinarians, social scientists, trained phlebotomists and field data collectors from the Institute of Epidemiology, Disease Control and Research (IEDCR), and the International Centre for Diarrhoeal Disease Research, Bangladesh (icddr,b) visited each outbreak community (defined as any community with at least one suspected cutaneous anthrax human case reported within 1 month of illness onset) at least twice and conducted investigations.

At the initial visit, trained field data collectors searched an outbreak community with a reported suspected cutaneous anthrax case to identify the implicated livestock exposure, i.e., the suspected livestock anthrax case(s). The team then searched each affected community to identify and list all persons exposed to each suspected livestock anthrax case in the previous 2 weeks from illness onset. The field or local health team followed up with each exposed individual for 2 weeks and categorized those with exposure and who developed symptoms suggestive of cutaneous anthrax as suspected cases.

Any person with acute skin lesion(s) developing over 2–6 days from a papular through a vesicular stage into a depressed black eschar with surrounding edema with or without fever, malaise, and lymphadenopathy having history of handling sick or slaughtered livestock (exposure to raw meat or body fluids or carcass or by-products) or contact with suspected livestock anthrax case(s) in the 2 weeks prior to illness onset was defined as a suspected case of cutaneous anthrax.

Any suspected cutaneous anthrax case with bacteriological evidence of *B. anthracis* infection (cultured cutaneous lesion swabs showing growth of characteristic, non haemolytic colonies; identification of Gram positive, non-motile, sporulated, bacilli on microscopy of culture smears stained with Gram stain and polychrome methylene blue; and lysed by gamma phage test) was defined as a laboratory confirmed cutaneous anthrax case (8, 17).

Any person with an illness clinically compatible with suspected cutaneous anthrax symptoms who was exposed in the previous 2 weeks before illness onset to raw meat, carcasses, body fluids or by-products of sick or dead livestock linked epidemiologically to a laboratory confirmed human or a confirmed livestock anthrax case was defined as a probable cutaneous anthrax case.

### Enrolment of participants for the case–control study

The field team members approached laboratory confirmed and probable cutaneous anthrax cases and sought voluntary informed written consent for participation in the case–control study. The team physicians conducted interviews to collect clinical and exposure information and clinically examined enrolled cases using a structured questionnaire. The location of each enrolled case’s household was recorded with a hand-held Global Positioning System. All persons from the outbreak communities who did not develop clinical illness within 2 weeks of exposure to raw meat, carcasses, body fluids or by-products of sick or dead livestock or suspected case(s) of livestock anthrax were listed as eligible controls. Suspected animal anthrax case was defined as a domestic ruminant with any of the following symptoms per World Organization for Animal Health/World Health Organization guidelines: a steep rise in temperature, dullness, shivering, difficulty breathing, collapse and convulsions, bloody discharge from the rectum or other natural openings and/or having sudden death within 24 h of onset of clinical illness, residing in the same village and with illness onset within 2 weeks of a laboratory-confirmed animal or human cutaneous anthrax case (8).

The study team recruited one eligible control from each of the four households nearest to but not in the household of an enrolled case. Enrolled controls were interviewed using the same structured questionnaire as enrolled cases, to collect exposure information.

### Sample collection and laboratory testing

Vesicular exudate swabs collected from skin lesions of affected persons were used for slide smear preparation. The slide smears were stained using Loeffler’s polychrome methylene blue for identification of anthrax bacilli by microscopy as described (18). IEDCR Microbiology laboratory performed staining and microscopy.

### Statistical analysis

We generated frequencies to describe the outbreaks in terms of person, place and time. To estimate the attack rate, we included only the 16 outbreaks where almost all individuals exposed to the suspected animal anthrax case(s) in the affected community could be reliably identified. We excluded the outbreaks from attack rate estimation if tracing and identification of all contacts to an implicated animal was unclear and/or incomplete. For the attack rate calculation, we specifically excluded 10 outbreaks that were reported from the district of Meherpur as we could not identify the total number of exposed individuals per implicated livestock. We divided the total number of suspected cutaneous cases by the total number of people who were exposed to raw meat, body fluids, carcass and/or by-products of a suspected livestock anthrax case(s) in the 2 weeks before outbreak onset to calculate the attack rate of suspected cases for each outbreak. We pooled these data across outbreaks to estimate the average proportion of exposed individuals developing the disease to determine the average attack rate. We also estimated age group specific attack rates.

We performed univariate analysis to identify the socio-demographic, occupational, nutritional, and behavioral risk factors. We estimated odds ratios (OR), 95% confidence intervals (CI) and significance levels (*p*-values) for individual risk factors. The variables with a *p*-value of <0.05 in univariate analysis were selected for multivariate analysis. Backward stepwise selection of variables with a significance level of 0.05 was used to construct final models. Variables with a *p*-value of >0.05 were removed from the model. We estimated the adjusted odds ratios (aOR) using multivariate logistic regression, combining all significant variables.

## Results

### Descriptive epidemiology, geographic distribution and seasonal trend

**Figure 2:**
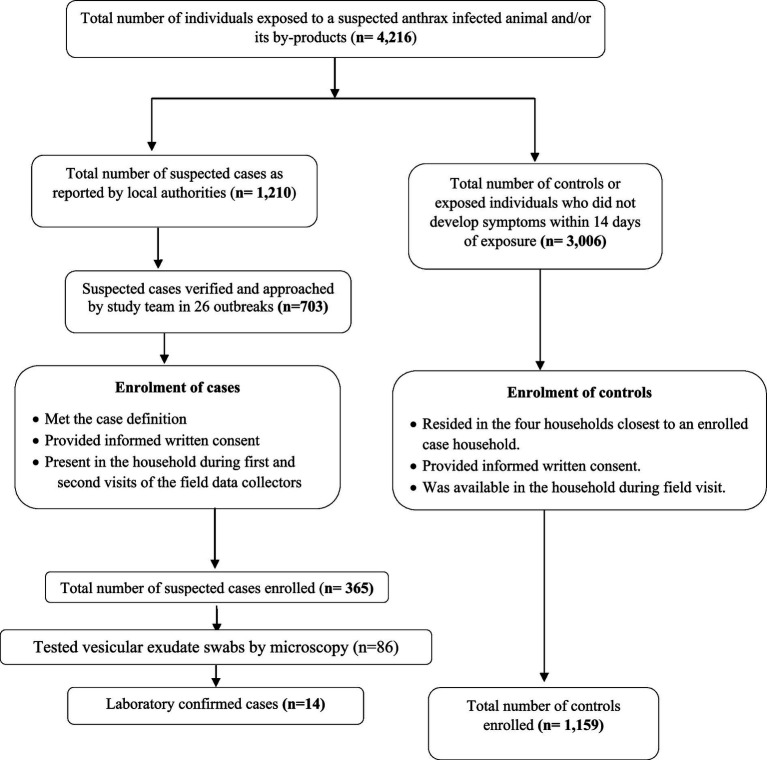
Flowchart showing participant enrolment into the anthrax case–control study from the 26 outbreaks reported in Bangladesh during 2013 and 2016.

**Figure 3:**
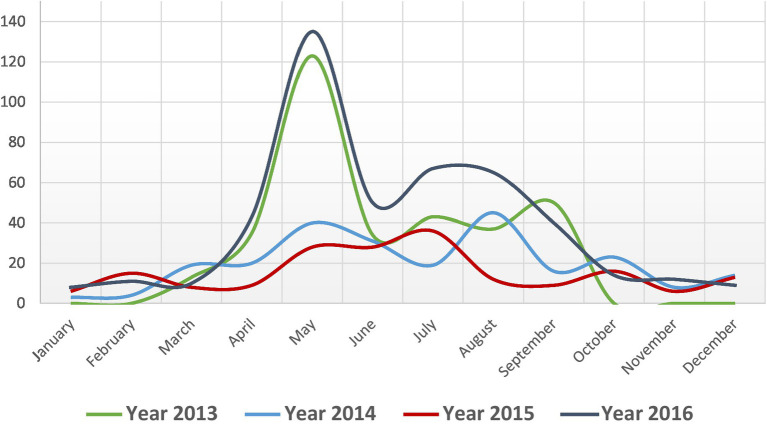
Monthly distribution of suspected cutaneous anthrax cases, Bangladesh, 2013–2016 (*n* = 1,210).

During 2013–2016, a total of 26 outbreaks were reported in seven districts of Bangladesh. A total of 1,210 suspected cutaneous anthrax cases were identified and the majority of the cases (61%, *n* = 744) were identified in the Meherpur district (Figure 2). The mean age of suspected cutaneous anthrax cases was 32 years (range: 1–84 years) and 50% were males. Human cutaneous anthrax cases were detected over the year, with the highest number of outbreaks occurred in May, typically increasing in the month of April and decreasing in October. This pattern was consistent during 2013–2016 (Figure 3). Cluster of human cutaneous anthrax cases reported from all seven districts except the border district of Meherpur where both sporadic and cluster of cases were reported throughout the year. Throughout the study period, no cases of gastrointestinal or inhalation anthrax were detected.

The overall attack rate of suspected cutaneous cases for 16 outbreaks was 20%. In these 16 outbreaks, a total of 2,121 individuals were identified as having a confirmed history of exposure to suspected animal anthrax cases. They had direct or indirect contact with suspected livestock anthrax case(s) and their by-products during handling, slaughtering, and processing. Among the exposed individuals, 421 (20%) developed skin lesions that progressed from a papular stage to a vesicular stage, eventually forming a depressed black eschar surrounded by edema. A total of 22 livestock (21 cattle and one buffalo) and their by-products were implicated in the 421 human cases of suspected cutaneous anthrax. The attack rates did not vary significantly by sex (21% in males and 19% in females). The attack rate was highest among people aged between 40 and 49 years and lowest among children aged below 10 years of age (Table 1). No death was reported during the study period.

**Table 1:** Attack rates of suspected cutaneous anthrax cases among those exposed to anthrax infected livestock by age group, Bangladesh, 2013–2016, (number of outbreaks = 16).

Age group	Total number of exposed individuals (*n* = 2,121)	Total number of suspected cases (*n* = 421)	Attack rate (%)
≤9 years	365	44	12
10–19 years	492	76	15
20–29 years	381	85	22
30–39 years	365	82	23
40–49 years	257	73	28
50–59 years	134	29	22
≥60 years	127	32	25

### Factors associated with suspected cutaneous anthrax

The study team visited the households of a total of 703 out of 1,210 probable and/or laboratory confirmed cutaneous anthrax cases for enrolment into the case–control study. Among the 703 cases approached during the field visit, 298 were absent from their households and 40 did not provide consent for enrolment into the study. Therefore, a total of 365 suspected cutaneous anthrax cases and 1,159 controls were recruited from five districts for the case–control study, following their informed written consent (Figure 2). The sociodemographic and clinical features of suspected 365 cases are presented in Supplementary Tables 1, 2. In univariate analysis, cases were more likely to have been exposed to the anthrax suspected livestock (OR 5.02, 95% CI: 3.16–7.96), had prior cut injuries on the exposed areas of the body (OR 33.53, 95% CI: 7.79–144.19), handled raw meat while butchering (OR 3.48, 95% CI: 2.72–4.44), slaughtered sick livestock (OR 6.59, 95% CI:4.55–9.55), weighed raw meat (OR 7.7, 95% CI: 4.71–12.59), assisted in skinning (OR 6.01, 95% CI: 3.09–11.69), mixed bones and meat together (OR 9.69, 95% CI: 6.63–14.15), skinned a dead livestock (OR 6.97, 95% CI: 3.96–12.25) and assisted in slaughtering (OR 7.89 95% CI: 4.87–12.78) than controls (Table 2).

**Table 2:** Univariate and multivariate analysis of risk factors for suspected cutaneous anthrax outbreaks, Bangladesh, 2013–2016.

Characteristics	Case *N* = 365 *n* (%)	Control *N* = 1,159 *n* (%)	OR (95% CI, *p*)	aOR (95% CI, *p*)
Direct exposure to the anthrax suspected livestock in the two weeks prior to the onset of outbreak				
No	21 (6)	272 (23)	Ref.	Ref.
Yes	344 (94)	887 (77)	5.02 (3.16–7.96, <0.001)	2.68 (1.61–4.45, <0.001)
Handled raw meat while butchering				
No	146 (40)	810 (70)	Ref.	
Yes	219 (60)	349 (30)	3.48 (2.72–4.44, <0.001)	
Slaughtered the sick livestock in the two weeks prior to outbreak onset				
No	295 (81)	1,108 (96)	Ref.	
Yes	70 (19)	51 (4)	6.59 (4.55–9.55, <0.001)	
Weighed raw meat				
No	312 (85)	1,134 (98)	Ref.	Ref.
Yes	53 (15)	25 (2)	7.7 (4.71–12.59, <0.001)	5.73 (3.03–10.83, <0.001)
Assisted in skinning				
No	340 (93)	1,145 (99)	Ref.	
Yes	25 (7)	14 (1)	6.01 (3.09–11.69, <0.001)	
Mixed bones and meat together				
No	264 (72)	1,115 (96)	Ref.	Ref.
Yes	101 (28)	44 (4)	9.69 (6.63–14.15, <0.001)	4.64 (3.03–7.09, <0.001)
Skinned dead livestock				
No	327 (90)	1,140 (98)		
Yes	38 (10)	19 (2)	6.97 (3.96–12.25, <0.001)	
Assisted in slaughtering				
No	309 (85)	1,133 (98)	Ref.	Ref.
Yes	56 (15)	26 (2)	7.89 (4.87–12.78, <0.001)	2.29 (1.3–4.02, 0.004)
Carried the stomach of slaughtered livestock				
No	335 (92)	1,132 (98)	Ref.	
Yes	30 (12)	27 (2)	3.75 (2.2–6.4, <0.001)	
Cleaned the stomach of slaughtered livestock				
No	325 (89)	1,103 (95)	Ref.	
Yes	40 (11)	56 (5)	2.42 (1.58–3.7, <0.001)	
Handling meat of sick slaughtered livestock during cooking				
No	261 (72)	905 (78)	Ref.	
Yes	104 (28)	254 (22)	1.4 (1.08–1.85, 0.01)	
Handling and washing meat of sick slaughtered livestock				
No	241 (66)	818 (71)	Ref.	
Yes	124 (34)	341 (29)	1.23 (0.96–1.58, 0.1)	
Observed livestock slaughtering				
No	267 (73)	1,048 (90)	Ref.	Ref.
Yes	98 (27)	111 (10)	3.46 (2.55–4.69, <0.001)	2.86 (2.02–4.04, <0.001)
Had a cut injury in the exposed parts of the body before handling raw meat of sick livestock				
No	345 (95)	1,157 (100)	Ref.	Ref.
Yes	20 (5)	2 (1)	33.53 (7.79–144.19, <0.001)	19.04 (4.08–88.86, <0.001)
Age of the respondents				
0–10 year	44 (12)	251 (22)	Ref.	
11–20 year	46 (18)	279 (24)	1.34 (0.88–2.04, 0.16)	
21–30 years	95 (26)	248 (21)	2.18 (1.46–3.25, <0.001)	
31–40 year	83 (23)	179 (15)	2.64 (1.75–3.99, <0.001)	
>40 year	77 (21)	202 (17)	2.17 (1.43–3.29, <0.001)	
Washed hands with soap and water after handling raw meat of sick/slaughtered livestock				
Yes	177 (49)	262 (22)	Ref.	Ref.
No	188 (52)	897 (77)	3.22 (2.51–4.12, <0.001)	2.57 (1.89–3.5, <0.001)
Washed hands with only water after handling raw meat of sick/slaughtered livestock				
Yes	195 (53)	343 (30)	Ref.	Ref.
No	170 (47)	816 (70)	2.72 (2.14–3.47, <0.001)	2.92 (2.18–3.91, <0.001)

Multivariate analysis identified several statistically significant risk factors for cutaneous anthrax. People who had a prior cut injury on the exposed areas of the body (aOR 19.04, 95% CI: 4.08–88.86), assisted in slaughtering (aOR 2.29, 95% CI: 1.3–4.02), had direct exposure to the anthrax suspected live livestock (aOR 2.68, 95% CI:1.61–4.45), weighed raw meat (aOR 5.73 95% CI: 3.03–10.83), mixed bones and meat together (aOR 4.64, 95% CI: 3.03–7.09), observed livestock slaughtering (aOR 2.86, 95% CI: 2.02–4.04), did not wash hands with soap and water after direct contact with suspected livestock or their byproducts (aOR 2.57, 95% CI: 1.89–3.5) and did not wash hands with water (aOR 2.92, 95% CI: 2.18–3.91) were more like to develop cutaneous anthrax than people who did not have these exposures (Table 2).

### Laboratory tests results

IEDCR microbiology laboratory tested vesicular exudate swabs from 86 cases; identified blue-stained bacilli with squared ends by microscopy of vesicular smears in 14 cases (16%).

## Discussion

Anthrax has been causing repeated outbreaks in both livestock and humans of Bangladesh (15, 19). Rural people were mostly affected because of frequent animal exposures and risky slaughtering practices (15). In this study, the outbreak investigation team identified multiple outbreaks in seven districts during 2013–2016 based on clinical, laboratory and epidemiological results. Majority of the anthrax outbreaks were reported from specific districts of north-western regions in Bangladesh where previous anthrax outbreaks had been reported, suggesting soil of these areas might contain more anthrax spores compared to other areas of Bangladesh (12, 15, 20, 21). An environmental study detected *Bacillus anthracis* spores in 14 soil samples from an anthrax outbreak district (Sirajganj) during January to November in 2012 (22). Anthrax outbreaks mostly occurred during the warm months following heavy rainfall which is supposed to accumulate spore in low-lying areas (23, 24). The spatial pattern of anthrax occurrences in certain regions may be linked to particular ecological factors that are known to facilitate the persistence and activation of spores. Alkaline soils containing abundant organic matter, calcium, and various minerals can promote the proliferation of anthrax spores (25). In contrast, a previous small-scale study from Bangladesh found anthrax spores most commonly in slightly acidic soil samples (6.38 ± 0.15) (22). In-depth future investigations examining the link between ecological parameter and occurrence of repeated anthrax outbreaks may provide insights into the ecological risk factors, which could be crucial for developing effective prevention and mitigation strategies.

Majority of the anthrax outbreaks occurred in Bangladesh during monsoon season (April–September) (12). In an earlier investigation conducted in Bangladesh, *Bacillus anthracis* isolates were identified in soil samples (11.67%) between May and November, while no isolates were found in samples collected during the dry season (December–April) (20). Heavy rainfall during monsoon after a long dry period and alkaline soil with adequate nitrogen promote the anthrax spore to become infective that resulted in outbreaks in livestock (22, 26). A study detected anthrax spore in loamy type soil samples suggesting soil type could influence the existence of anthrax (22). Though our study detected human cases throughout the year, the highest number of outbreaks occurred in May, with the majority detected between April and October, which aligns closely with previous reports.

The attack rate of suspected cutaneous cases estimated in this study was 20% among the exposed individuals which was very similar to the attack rate (21.1%) reported by a study from Kenya (27). A previous study from Bangladesh reported a higher attack rate (49.2%) among individuals who slaughtered sick animals (12). An outbreak investigation in India reported relatively lower attack rate (7%) but the case fatality rate was higher (18%) (28). Untreated cutaneous anthrax can be fatal in up to 1 in 5 people (17). No fatal cases were reported in this study, possibly attributed to efficient healthcare access, prompt initiation of antibiotic therapy and cutaneous form of anthrax. Most of the previous human anthrax outbreaks caused cutaneous type lesions and almost all human cases were associated with animal cases (12, 15, 20, 21). We also found similar clinical features of affected cases in this study. Similarly, all affected cases were exposed to anthrax suspected livestock and/or their by-products either directly or indirectly. The lower attack rate detected in this study compared to previous outbreaks in Bangladesh could be due to prior infection or exposure to anthrax and its associated acquired immunity. A previous study in the Kayseri region of Turkey detected T cell memory such as CD4 T cell response in humans after several years of infection (29). People of all age groups were susceptible to develop infection but people aged between 40 and 49 years were mostly infected in this study. A previous study reported higher infection rates in people aged between 21 and 30 years (12). On the contrary, one study reported lowest attack rates (7.1 cases per 10,000 persons) among people aged 15–44 years of age (30). As adults and younger individuals were likely to be mainly engaged in animal slaughtering, they may be expected to be more vulnerable to developing infection because of occupational exposure.

We identified several risky practices for human anthrax infection. The occurrence of anthrax infection in humans was significantly linked to prior cut injuries on exposed body parts, direct exposure to suspected anthrax-infected animals and their by-products, as well as involvement in slaughtering activities. Similar risky practices were identified by previous studies from Bangladesh (12, 15). Most of the livestock farmers have low-moderate income and they are at high risk for contracting anthrax because of frequent interaction with livestock. Their family income partially comes from animal husbandry. Slaughtering sick livestock is commonly practiced in rural areas of Bangladesh reported by a few studies (15, 31, 32). Though the price of meat from a sick animal is usually lower than a healthy animal, farmers try to overcome financial loss by selling meat from sick animals. Poor people living in rural areas prefer to buy meat from sick animals at a lower price. This kind of practices contributed to *Bacillus anthracis* transmission from affected animals to humans. When a sick animal is slaughtered, many people living in the community are exposed to affected animals by slaughtering, skinning and processing meat (12). Regardless of profession, age, or gender, individuals in the communities, including farmers and butchers, engage in slaughtering and processing meat and other animal by-products (15). People with abrasions, cuts and fissures in their skin are more likely to be at high risk for getting anthrax infection that other people (33). Individuals with pre-existing cut injuries on exposed areas of their bodies were also found to be more susceptible to infection in this study. Slaughtering, skinning and processing meat are laborious works that expose individuals to injury risks. Though many people are exposed to anthrax suspected sick animals, a portion of individuals developed cutaneous anthrax lesions. The presence of cut injury could be the main contributing factor for getting infection from infected livestock. Inadequate livestock vaccine, low vaccination coverage, poor awareness about the impact of regular vaccination and access of high-quality vaccines may contribute to the occurrence of anthrax infection in livestock (12, 34). Mass awareness of the livestock farmers and community people regarding the risk of slaughtering sick livestock, importance of anthrax vaccination and deep burial practices for deceased livestock can be effective in controlling anthrax in anthrax prone areas.

This study revealed that handwashing with soap following direct contact with sick or slaughtered livestock or their byproducts was more likely to protect the transmission of *Bacillus anthracis* from infected livestock to human. Handwashing was found effective against foodborne diseases and respiratory infections worldwide (35). Handwashing with soap can interrupt transmission of infectious diseases and can act as a type of environment decontaminant (36). Most of the farmers living in rural areas belong to the low to middle-income class and they often slaughter moribund livestock to minimize financial loss due to the dead of the livestock. Stopping slaughtering sick animals may not be feasible due to the economic repercussions stemming from the loss of these animals particularly in rural areas. There is no functional system of financial incentives or health insurance for the poor-middle income farmers in Bangladesh. Given this scenario, focusing on implementing handwashing with water and/or soap after direct contact with sick livestock and their by-products within the affected communities could help mitigate certain risks.

This study has few limitations. The cases and controls were mostly selected based on clinico-epidemiological evidence. Although controls were selected from those who were exposed to sick and/or slaughtered animals and/or their by-products, there may be possibility of latent or mild infections. This suggests the possibility of some misclassification bias in selection of controls in this case–control analysis. The laboratory diagnosis of cases in this study was primarily relied on staining by Loeffler’s polychrome methylene blue and microscopy, although the identification of *B. anthracis* via microscopy was considered presumptive. Due to limited resources, we were unable to use real-time polymerase chain reaction (RT-PCR) to confirm cases.

## Conclusion

*Bacillus anthracis* caused recurrent outbreaks affecting both humans and livestock in the northwestern part of Bangladesh. The soil in this region might harbor anthrax spores more abundantly compared to other parts of the country. Stopping slaughtering sick livestock, handling meat and animal by-products to reduce anthrax exposures from livestock to humans may be difficult to achieve given the associated financial incentives in Bangladesh. Interventions such as hand washing with water and/or soap after the contact with sick animals and their by-products can be targeted to affected communities to ameliorate some risk. The government should prioritize annual vaccination of all cattle and goats in this region to reduce livestock outbreaks and promote safe burial of dead livestock, minimizing the spillover of *Bacillus anthracis* into the environment.

## Data Availability

The raw data supporting the conclusions of this article will be made available by the authors, without undue reservation.
