# Supplementary material for: Risk factors associated with cutaneous anthrax outbreaks in humans in Bangladesh
Source: Front Public Health. 2024 Oct 15;12:1442937. doi: 10.3389/fpubh.2024.1442937 (PMC11518833; doi:10.3389/fpubh.2024.1442937)
Supplement: Supplementary file 1 [file Table_1.docx]

**Supplementary Table 1: Demographic characteristics of suspected cutaneous anthrax cases enrolled from five districts for the case-control study, 2013−2016 (n=365)**

| **Demographic characteristics** |  |
| --- | --- |
| **Median age in years, (Inter-quartile range, IQR)** | 30 (17-40) |
| **Gender, n (%)**  **Female**  **Male** | 182 (50)  183 (50) |
| **Occupation, n (%)** |  |
| Housewife | 107 (30) |
| Agriculture worker | 76 (21) |
| Student | 79 (22) |
| Businessman | 18 (5) |
| Day laborer | 16 (4) |
| **Outbreak districts, n (%)** |  |
| Meherpur | 90 (25) |
| Kustia | 19 (5) |
| Rajshahi | 20 (5) |
| Sirajganj | 140 (38) |
| Tangail | 96 (26) |
| **Year of detection, n (%)** |  |
| 2013 | 72 (20) |
| 2014 | 103 (28) |
| 2015 | 39 (11) |
| 2016 | 151 (41) |
