# Supplementary material for: Risk factors associated with cutaneous anthrax outbreaks in humans in Bangladesh
Source: Front Public Health. 2024 Oct 15;12:1442937. doi: 10.3389/fpubh.2024.1442937 (PMC11518833; doi:10.3389/fpubh.2024.1442937)
Supplement: Supplementary file 2 [file Table_2.docx]

**Supplementary Table 2: Clinical features of suspected cutaneous anthrax cases enrolled into the case-control study, Bangladesh, 2013−2016 (n=365)**

| **Clinical presentation** | **Number (%)** |
| --- | --- |
| **Skin lesion** | 335 (92) |
| **Site of skin lesion** |  |
| Upper limbs | 212 (58) |
| Lower limbs | 50 (14) |
| Face | 24 (7) |
| Neck | 11 (3) |
| Abdomen | 6 (2) |
| Chest | 6 (2) |
| Axillary | 90 (25) |
| Inguinal | 30 (8) |
| Cervical | 36 (10) |
| **Central black Eschar** | 255 (70) |
| **Vesicle** | 173 (48) |
| **Papule** | 163 (45) |
| **Ulcer** | 192 (53) |
| **Itching around skin lesion** | 119 (33) |
| **Surrounding edema** | 197 (54) |
| **Surrounding erythema** | 115 (32) |
| **Tenderness on palpation** | 157 (43) |
| **Fever** | 222 (61) |
| **Severe tiredness** | 52 (14) |
| **Headache** | 59 (16) |
| **Abdominal pain** | 20 (6) |
| **Vomiting** | 22 (6) |
| **Anorexia** | 44 (12) |
| **Nausea** | 27 (7) |
| **Neck pain** | 11 (3) |
| **Hypoxia** | 6 (2) |
| **Sore throat** | 14 (4) |
| **Chest pain** | 13 (4) |
| **Irritability** | 13 (4) |
| **Diarrhoea** | 8 (2) |
