# Supplementary figures and images for: Risk factors associated with cutaneous anthrax outbreaks in humans in Bangladesh
Source: Front Public Health. 2024 Oct 15;12:1442937. doi: 10.3389/fpubh.2024.1442937 (PMC11518833; doi:10.3389/fpubh.2024.1442937)

**Supplementary Figure 1:** Information sources for notification of cutaneous anthrax outbreaks


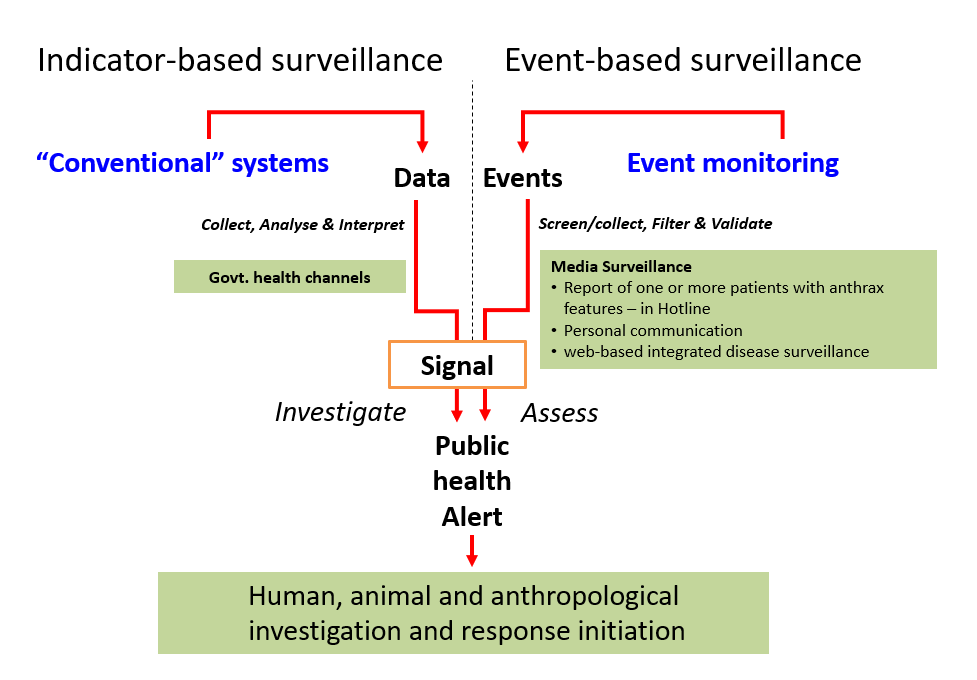

Supplement: Supplementary file 3 [file Data_Sheet_1.docx]
